# Next-Generation Sequencing Reveals the Total Yeast and Mold Species Present in Greenhouse-Grown Cannabis Inflorescences—Implications for Quality Control

**DOI:** 10.3390/plants15213339

**Published:** 2026-10-28

**Authors:** Zamir K. Punja

**Affiliations:** Department of Biological Sciences, Simon Fraser University, 8888 University Drive, Burnaby, BC V5A 1S6, Canada; punja@sfu.ca

**Keywords:** plant microbiome, soil microbes, plant pathogens, microbial diversity, yeasts and molds

## Abstract

Next-generation sequencing (NGS) was conducted to determine the composition of the fungal and yeast species (mycobiome) within inflorescences of seven THC-containing genotypes of cannabis (*Cannabis sativa* L.). They were grown under conventional hydroponic cultivation or under organic production in commercial greenhouses. Up to 26 fungal and yeast species were identified from DNA sequence homologies to representative species in databanks. The mycobiome composition varied according to cultivation method, cannabis genotype, and post-harvest drying. Highest numbers of total fungal reads were present in organically grown inflorescences. Stem tissues supporting the inflorescences contained up to 50% of total fungal reads. Using scanning electron microscopy, fungal spore-like structures and mycelium were observed within the stem tissues. The genera represented by >1% of total fungal reads within inflorescences were *Alternaria*, *Aspergillus*, *Botrytis*, *Candida*, *Fusarium*, *Penicillium*, *Trichoderma* and *Rhizophagus*. Post-harvest drying of the inflorescences reduced total fungal reads by 50–65%, depending on cannabis genotype and fungal species. Detached trichome glands subjected to NGS analysis revealed a range of air-disseminated fungi, including *Penicillium*, *Fusarium*, *Beauveria*, *Trichoderma*, *Aspergillus*, *Botrytis*, *Alternaria* and *Aureobasidium*. Spore-like structures were observed attached to the external surfaces of glandular trichomes using scanning electron microscopy. The occurrence of mycotoxin-producing species of *Aspergillus* was rare, while species of *Alternaria*, *Fusarium* and *Penicillium*, recognized as potential mycotoxin producers, were more frequent. The most prevalent fungal species reads among 12 dried cannabis samples were *Penicillium chrysogenum*, *P. citrinum* and *Alternaria* spp.; *Botrytis* and *Fusarium* spp. were also present in six samples. The use of biological control products containing *Trichoderma* and *Beauveria* during flower development increased fungal reads of these species in fresh inflorescences, with some carry-over into dried samples. The mycorrhizal fungus *Rhizophagus irregularis* was detected in inflorescence tissues. Reads of a number of previously unreported fungal species were present within inflorescences at very low frequencies. Their origins may be from the surrounding environment or the growing substrate used during cannabis cultivation. Human DNA was found in a number of dried samples, possibly from physical handling of post-harvest inflorescences. These results indicate the diversity and prevalence of certain genera of fungi/yeasts in greenhouse-cultivated cannabis inflorescences.

## 1. Introduction

**Figure 1:**
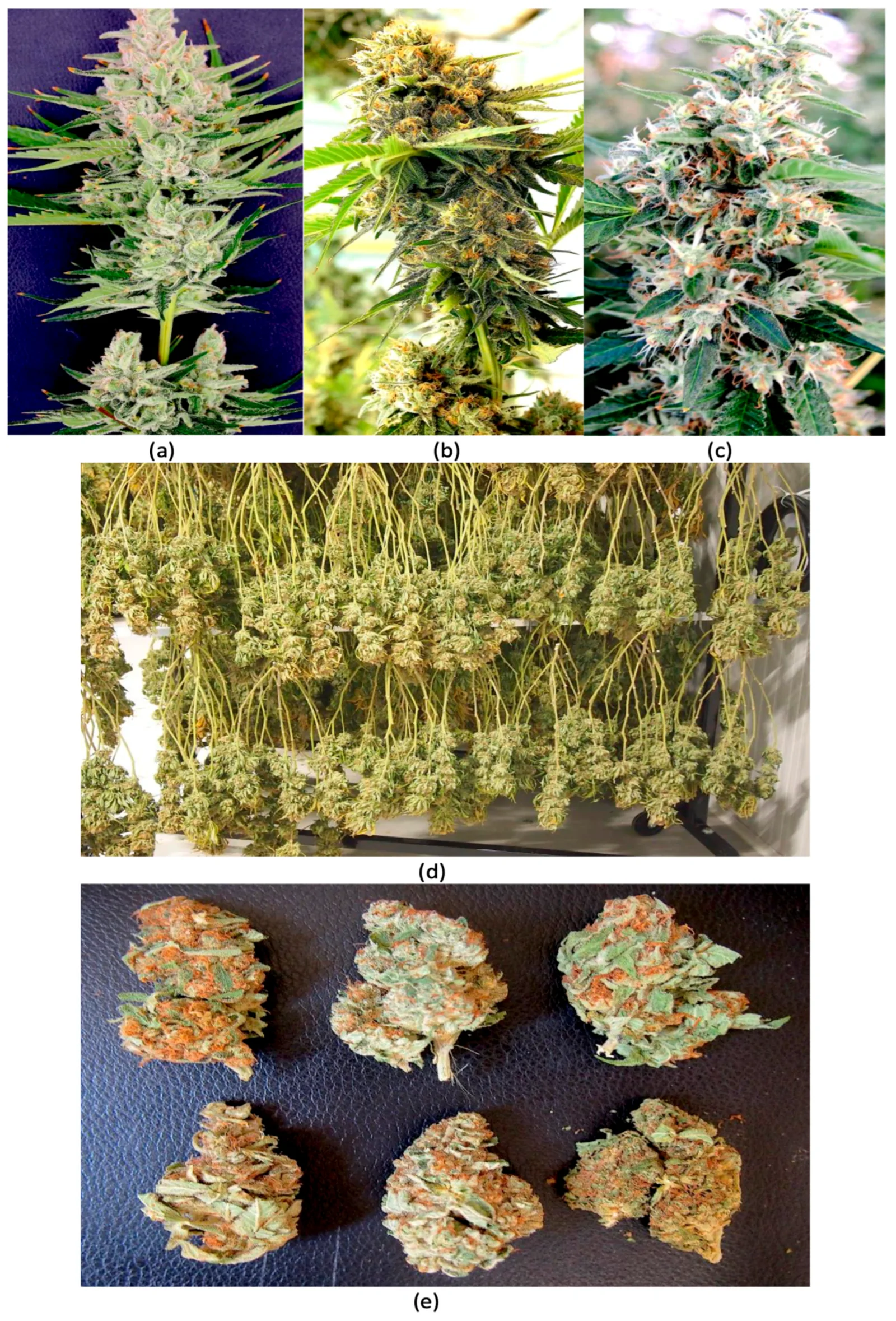
(**a**–**c**) Inflorescence structures of three cannabis genotypes included in this study. (**d**) The method of hang drying following which samples were obtained for microbiome analysis. (**e**) Dried and trimmed buds of six genotypes included in this study for microbiome analysis.

Cannabis (*Cannabis sativa* L.) containing high levels of delta^9^-tetrahydrocannabinol (THC) is grown commercially in Canada, in many regions of the USA, and in other parts of the world where regulatory approval in the appropriate jurisdictions has been granted. The cannabis plants are grown for their large inflorescence structures (Figure 1a–c), which are harvested and dried (Figure 1d) and subsequently distributed to medicinal and recreational sectors. The most common products reaching the market are components of the dried inflorescences or “buds” (Figure 1e).

The majority of commercial production of cannabis occurs within greenhouses or indoor growing rooms, where environmental conditions, lighting, and nutrient regimes can be controlled. Under the high plant densities in these growing environments, however, a major challenge is the management of various plant pathogens, including fungi, bacteria, viruses and viroids, which can appear and spread rapidly on cannabis plants [1,2,3,4]. In addition to reducing the yield and quality of affected plants, these pathogens can also directly infect the inflorescences, causing significant losses due to “bud rot” [5,6]. The carry-over of pathogens such as *Botrytis* and *Fusarium* species, for example, in dried cannabis products highlights concerns for cannabis users on the possible harm from inhalation of spores or mycotoxins that may be present [7]. Consequently, dried cannabis buds intended for human use must be tested for the presence of total yeasts and molds, specific bacterial species that are known human pathogens, and levels of specific mycotoxins, pesticides and other contaminants [8,9,10]. 

The testing of cannabis products for total yeast and mold levels (TYM) is conducted by commercial laboratories using several methods to establish final culturable species on agar media or membranes, and are expressed as colony-forming units (cfu) per gram of dried product [11,12,13]. These levels must fall below the thresholds established in the individual jurisdictions where cannabis is grown and are intended to provide a safety level for consumers. In Canada, the maximum limit is 50,000 cfu/gm; in the USA, individual states have established specific regulatory levels beyond which the product, if it exceeds the limit, cannot be sold [14]. Therefore, understanding the sources of TYM within a cannabis greenhouse or a specific growing environment is critical to avoid potential failure of the product. In addition, the fungal and yeast species that may be present can provide useful information to establish their sources and the degree to which they may (or may not) cause harm to cannabis users. However, the current assays utilized to determine culturable TYM levels that result in the acceptance/rejection of a cannabis product intended for human use may or may not be representative of the complete spectrum of the mycobiome present in that sample. This raises the question of whether the tests are providing the perceived safety assurance of the product. A few previous studies have attempted to identify the yeasts and molds present in cannabis tissues to genus or species level to determine their potential for harm if consumed by cannabis users [15,16,17,18,19,20].

In addition to the use of culture media to assess TYM, molecular detection methods based on the polymerase chain reaction (PCR) can provide rapid and accurate assessment of specific species that may be present in cannabis samples [20]. As well, whole-genome sequencing (next-generation sequencing, NGS) can be utilized to identify the microbial species which are present in a sample, including those that cannot be cultured on agar media [16,17,18,19]. This approach was used to determine the occurrence of fungal and yeast species present as endophytes in vegetative tissues of greenhouse-grown cannabis plants [21]. It was reported that certain endophytic yeast and mold species were distributed in mother plants, vegetative cuttings derived from them, and in flowering plants of cannabis. However, the extent to which these species may be found in inflorescence tissues has not been previously determined. 

**Figure 2:**
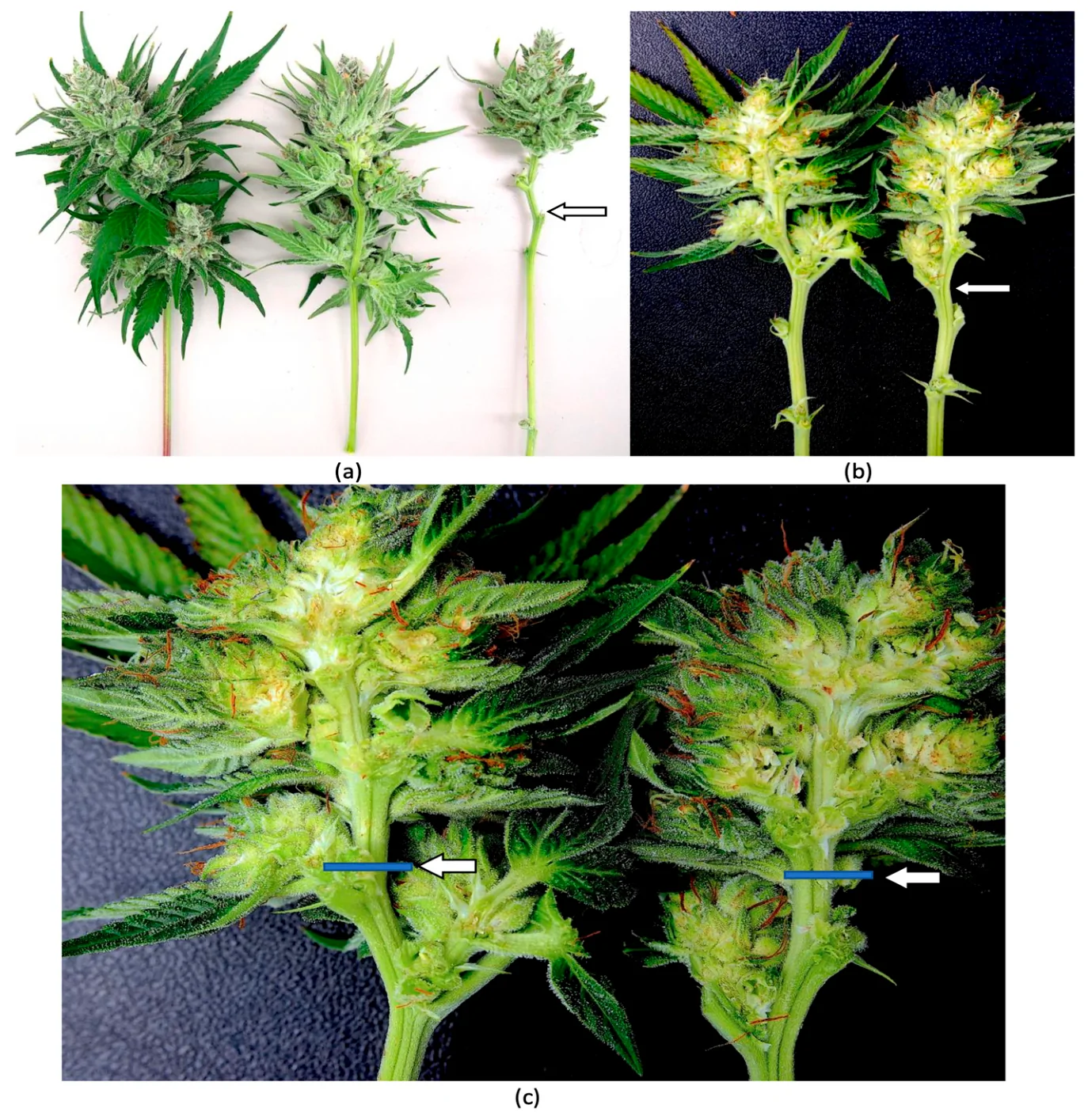
(**a**,**b**) The inflorescence stem (arrow) was sampled for microbiome analysis. (**c**) Cross-sections of the inflorescence stem (arrows) were obtained for microscopic studies.

**Figure 3:**
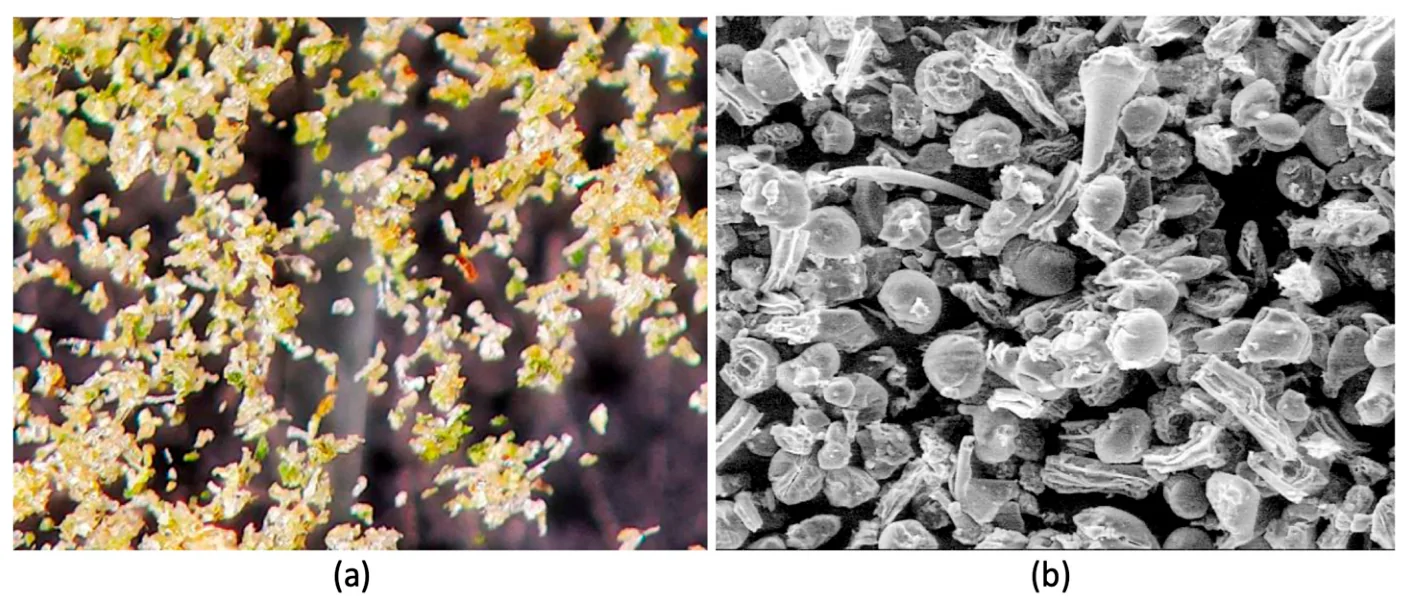
(**a**,**b**) Trichome preparations included for microbiome analysis by NGS. (**a**) Light microscope image under a dissecting scope. (**b**) Scanning electron microscope image of trichome glands.

An NGS approach using shot-gun cloning was used to establish the yeast and mold species comprising the mycobiome in cannabis inflorescences in the present study, providing a unique glimpse into their source(s) and how they may vary by genotype, post-harvest drying and production method (conventional or organic). Whole inflorescence samples as well as stems supporting the inflorescence (Figure 2), and trichome preparations (Figure 3) were used as starting materials for extraction of DNA. In addition, corresponding tissues were examined using scanning electron microscopy to identify sites where TYM may be found within the inflorescence tissues. The results presented demonstrate how next-generation sequencing and microscopic approaches can yield important findings regarding the composition of TYM in cannabis inflorescences.

## 2. Results

### 2.1. Sample Sources

Inflorescences of greenhouse-grown cannabis plants that were selected for sampling in this study are shown in Figure 1a–c and represent three of the genotypes sampled. These inflorescences were processed for NGS analysis using fresh tissues as well as dissected fresh stem tissues supporting the inflorescences (Figure 2a–c). The tissues were used without surface-sterilization. Following harvest and drying according to commercial procedures (Figure 1d), samples of the same dried inflorescences were also subjected to NGS analysis following DNA extraction. The appearance of six of the genotypes included in this study is shown in Figure 1e. The trichome preparation included for NGS analysis is shown in Figure 3a,b. A sample of 50 mg was used for DNA extraction.

### 2.2. Next-Generation Sequencing

**Figure 4:**
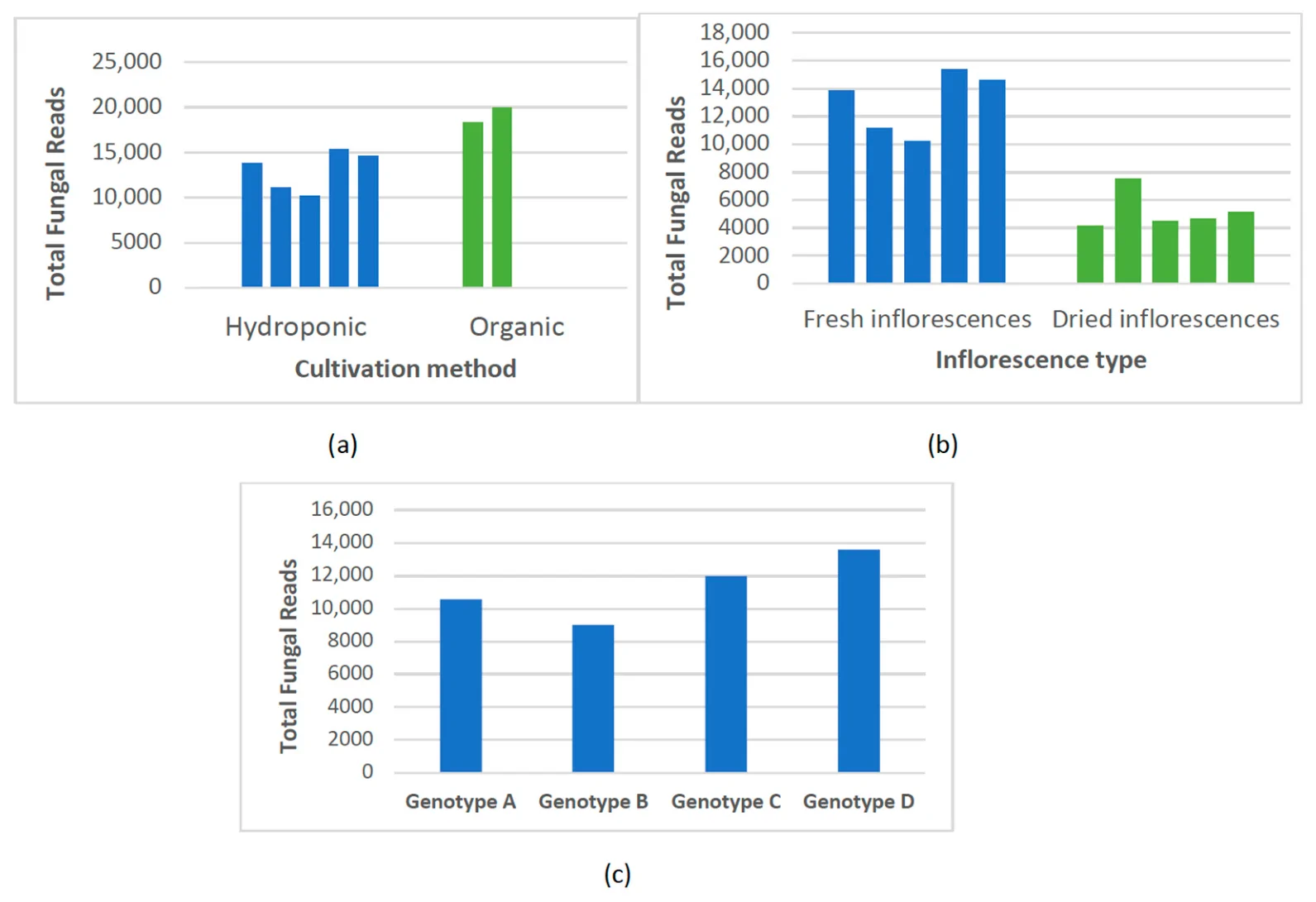
Total fungal reads following whole genome sequencing of samples of cannabis inflorescences from various sources included in this study. (**a**) A comparison of hydroponically (5 samples) and organically cultivated (2 samples) of fresh inflorescences. (**b**) A comparison of fresh and dried inflorescence samples from hydroponic cultivation. (**c**) A comparison of four different genotypes from hydroponic cultivation. All samples represent single replicates obtained from bulked samples from individual plants.

**Figure 5:**
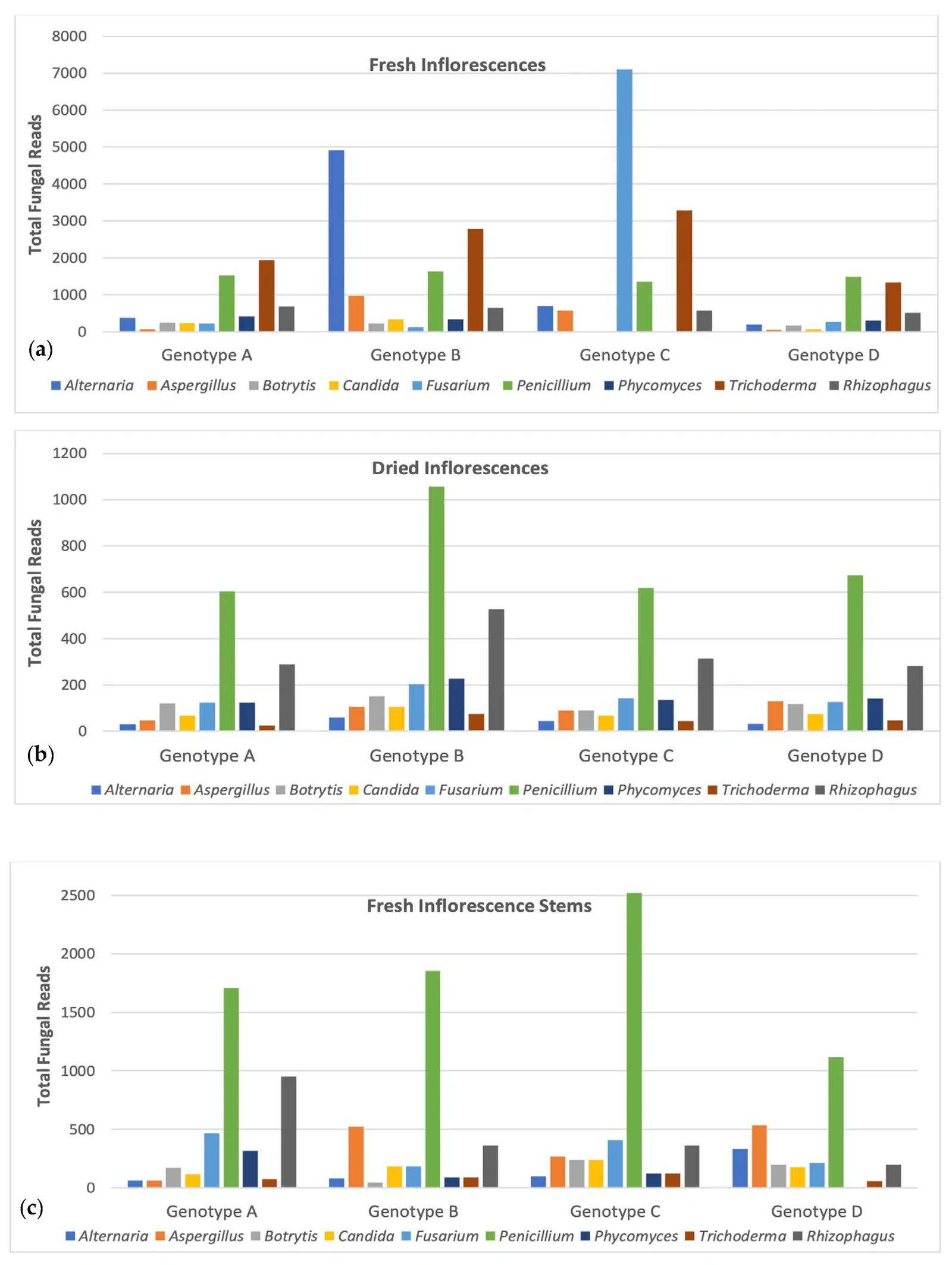
Total fungal reads of specific genera of fungi and yeasts that were most predominant in hydroponically cultivated cannabis inflorescence samples from four different genotypes. The same nine genera of fungi/yeasts are shown on each graph. (**a**) Fresh inflorescence samples. (**b**) Dried inflorescence samples. (**c**) Fresh inflorescence stem samples.

The total fungal sequencing reads and assigned species presented as Krona plots for each of the 24 inflorescence samples included in this study can be found at https://kannapedia.net/strains and are summarized in Supplementary Table S1. In addition, several samples of seed and growing substrate were included in the analysis. The results for inflorescence samples are presented below as either total fungal reads (inclusive of yeasts) in each of the respective samples (Figure 4 and Figure 5) or as a percentage of the total fungal reads (inclusive of yeasts) present in each sample (Table 1 and Table 2). These samples represent single replicates. The effect of production systems (hydroponic vs. organic) on total fungal reads is shown in Figure 4a. Organically produced samples (two samples) had higher overall fungal reads compared to hydroponically produced samples (five samples), although statistical inferences cannot be made due to the limited sample size. Drying of the samples reduced total detectable fungal reads in the five genotypes by around 50–65% (Figure 4b). The effect of cannabis genotype on total fungal reads is shown in Figure 4c. The genotypes varied in the total fungal reads present. These data were obtained from single samples collected from bulked flower samples from each plant.

The genera of fungi and yeasts that occurred at the highest frequencies among the total fungal reads in all cannabis genotypes were *Alternaria*, *Aspergillus*, *Botrytis*, *Candida*, *Fusarium*, *Penicillium*, *Phycomyces*, *Trichoderma* and *Rhizophagus* (Figure 5). The total fungal reads of these genera in fresh inflorescence samples of four cannabis genotypes are shown in Figure 5a. *Alternaria* and *Fusarium* were predominant in genotypes B and C, respectively, and were less abundant in genotypes A and D. *Trichoderma* was the next most abundant genus and was present in fresh inflorescence samples of all four genotypes. The next most abundant genotype was *Penicillium*, followed by *Rhizophagus*, in fresh inflorescences (Figure 5a). When the inflorescences were dried, there was a reduction in the total fungal reads observed, and the predominant genus present in all four genotypes was *Penicillium* (Figure 5b). The next most abundant genus was *Rhizophagus*, which was present in all samples. The remaining genera were present at much lower levels (< 200 reads). In fresh inflorescence stem samples, the most prevalent genus was *Penicillium*, followed by *Aspergillus* and *Rhizophagus* (Figure 5c). The remaining genera were present at lower levels (<500 reads).

The fungal reads of the most prevalent genera and species of fungi and yeasts present in all of the tissue samples analyzed in the study are shown in Table 1. These genera were present at frequencies >1% of the total fungal reads. The data presented are the averages across four genotypes. They include all the genera shown in Figure 5. Up to 26 species were identified in fresh and dried cannabis inflorescences. Among these, *Candida* was the only yeast species present at a frequency >1%. Presence of the species in samples of seed and growing medium was confirmed where available.

**Figure 6:**
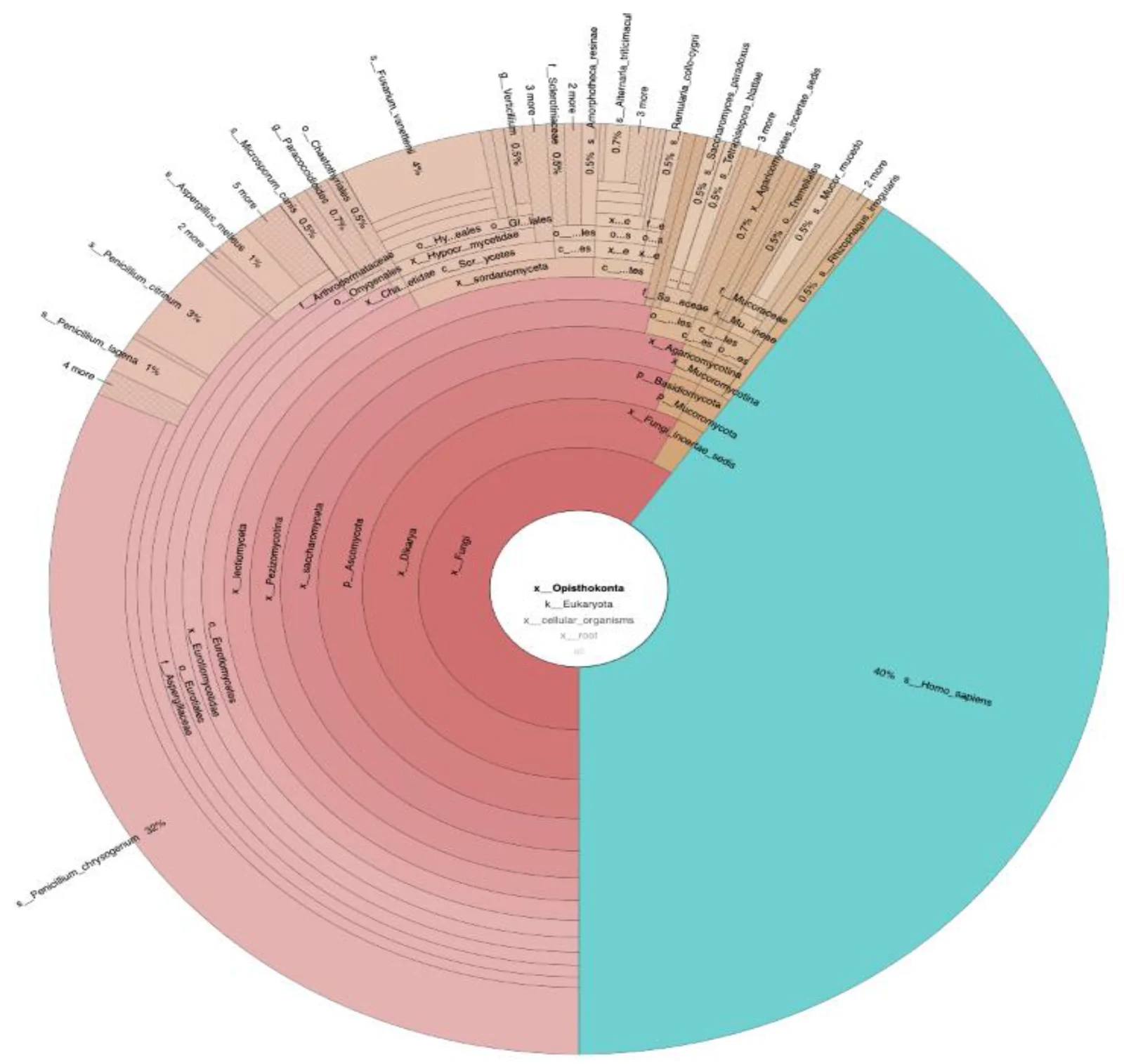
A Krona plot of a dried cannabis sample showing the occurrence of a number of species of fungi (brown-shaded area) as well as of human DNA (blue-shaded area).

In Table 2, the occurrence of a range of fungal, yeast and other organisms that were present in fresh and dried cannabis inflorescences is shown. The incidence of these species was less than 1% of the total fungal reads. They are presented here to demonstrate the occurrence of a range of minor or secondary species in addition to the primary species shown in Table 1. Fresh inflorescences had a greater diversity of species present, while drying reduced the numbers of species that were present. In addition to fungi and yeasts, two species of lichens (*Endocarpon* and *Letharia*) were identified in the samples. Of interest was the presence of DNA that matched that of humans (*Homo sapiens*), which was present in dried cannabis inflorescences but not in fresh samples (Table 2). This suggests that contamination may have resulted from the handling and processing of the dried samples after harvest by workers or possibly during DNA extraction of the samples (Figure 6). 

### 2.3. Scanning Electron Microscopy

**Figure 7:**
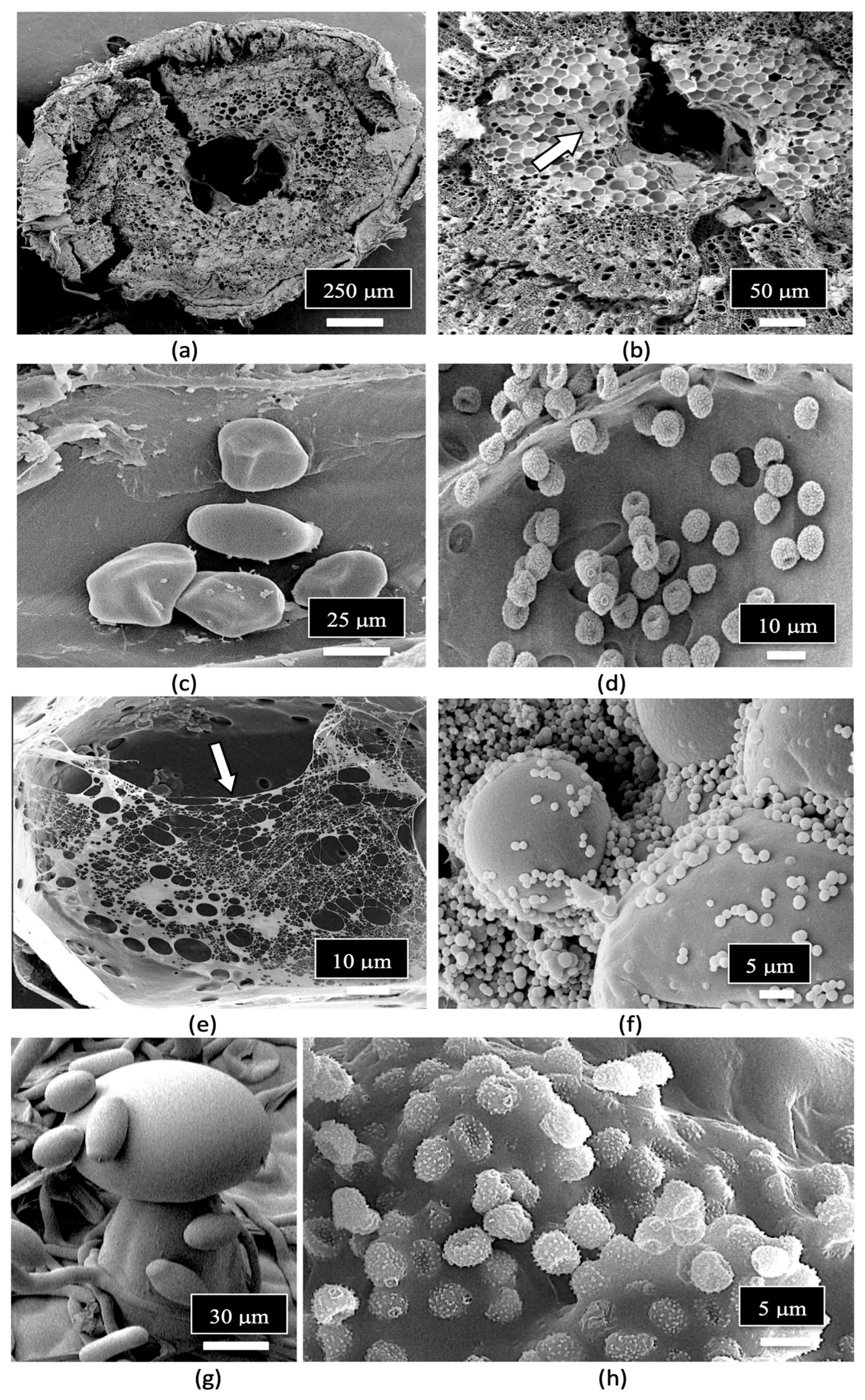
Scanning electron microscopic images of the inflorescence stems and trichomes of cannabis inflorescences and associated fungal-like spores and mycelium. (**a**) Section through an inflorescence stem showing the arrangement of cell layers around the central pith. (**b**) A close-up image of the cell layers surrounding the pith. Arrow points to the pith parenchyma cells. This cell layer is surrounded by the xylem vascular tissues. (**c**,**d**) Spores of fungal-like structures observed within the cells of the xylem tracheids. (**e**) Mycelium growing across a tracheid cell. (**f**) Spores of an unidentified fungal/yeast species adhered to the surface of glandular trichomes. (**g**) Spores of the powdery mildew fungus adhered to the surface of a glandular trichome. (**h**) Spores of probable *Penicillium* sp. embedded in the resin produced from glandular trichomes.

**Figure 8:**
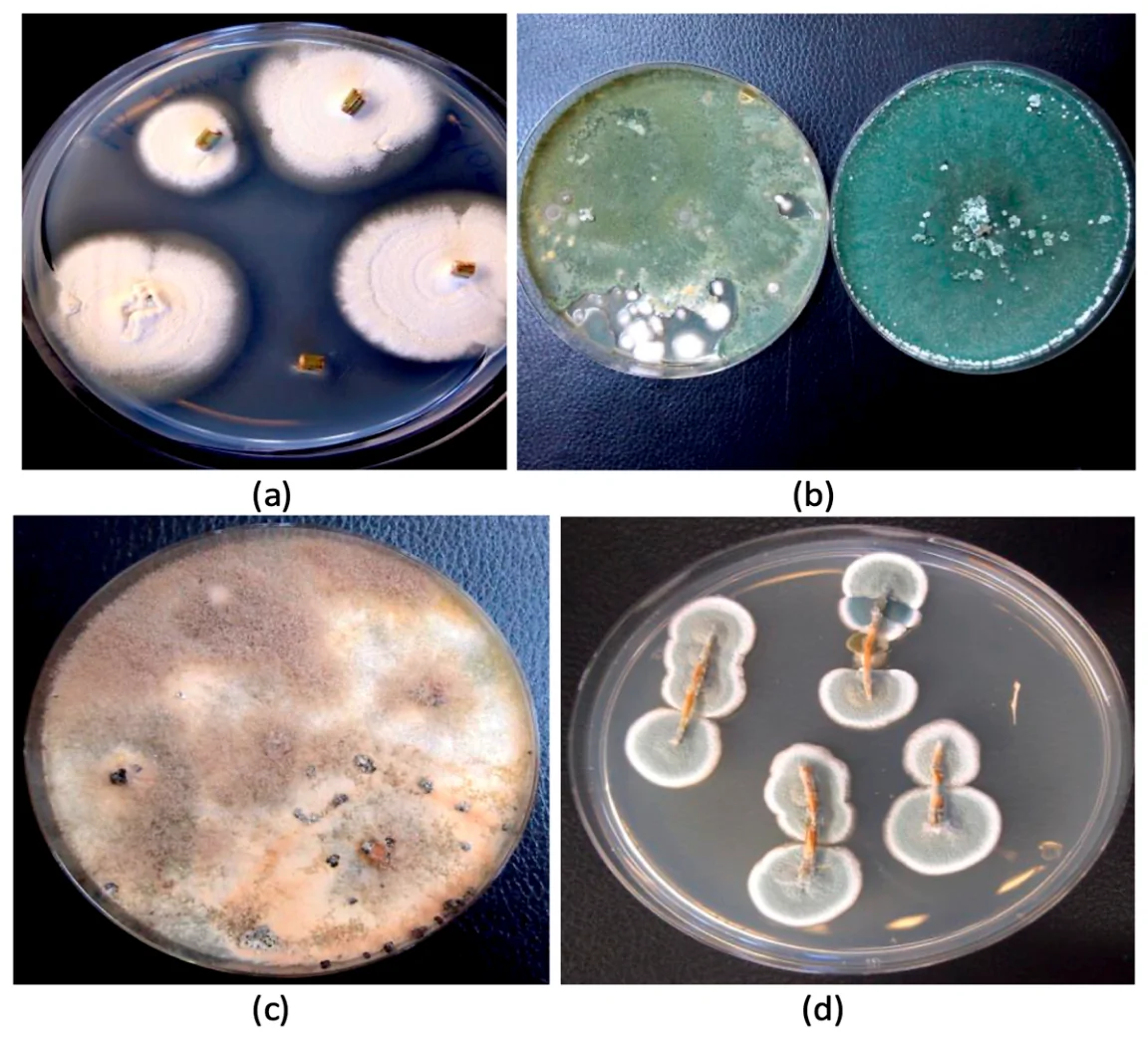
Recovery of several fungi from inflorescence stems of cannabis plants after plating surface-sterilized segments on potato dextrose agar. (**a**) *Beauveria bassianae.* (**b**) *Trichoderma harzianum*. (**c**) *Botrytis cinerea*. (**d**) *Penicillium chrysogenum*. Photos show growth after 8 days at 25 °C.

**Figure 9:**
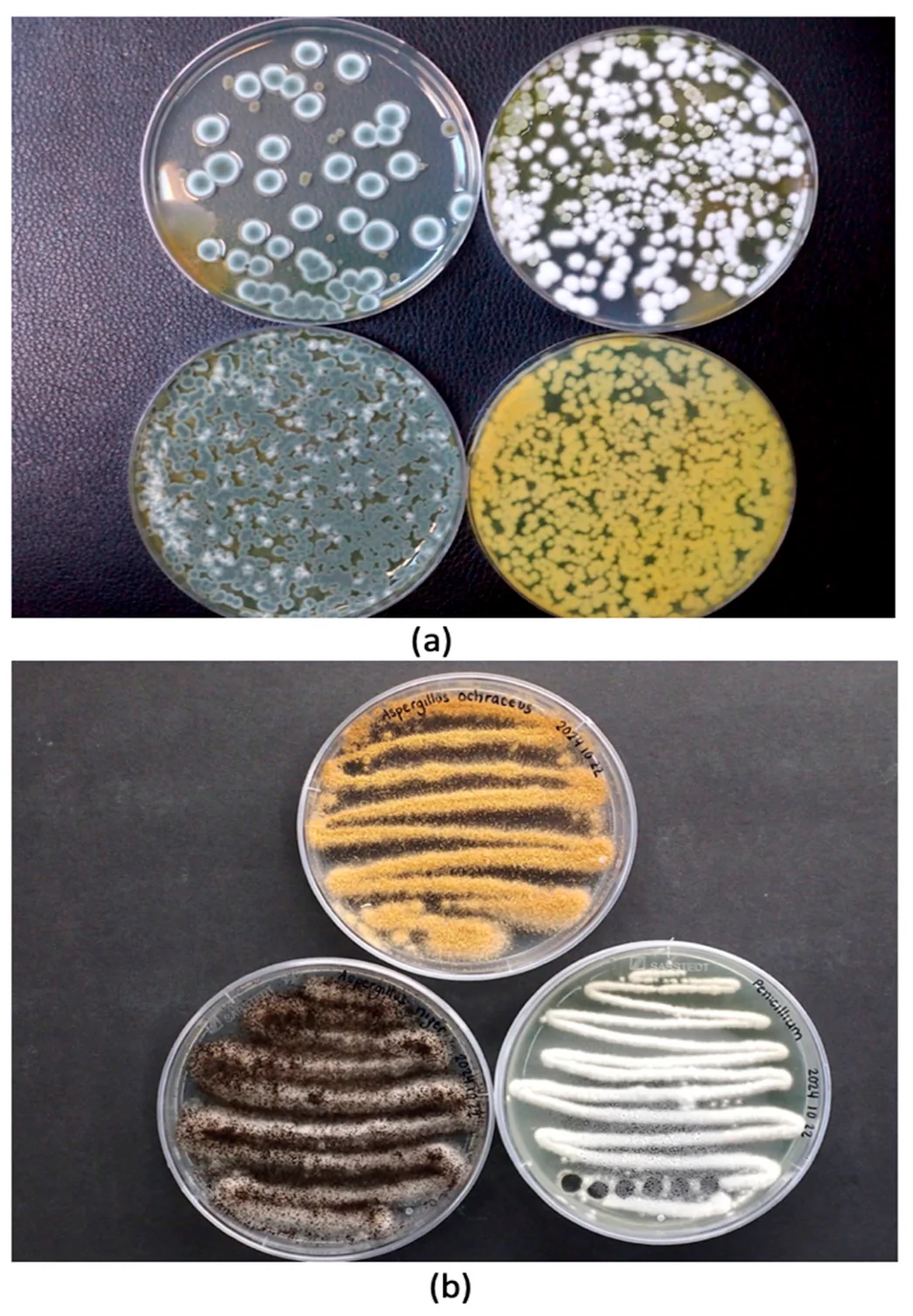
The species of *Penicillium* (**a**) and *Aspergillus* (**b**) recovered from dried greenhouse-grown cannabis inflorescences plated onto potato dextrose agar with the antibiotic streptomycin sulfate. (**a**) *Penicillium chrysogenum* (top left) *Penicillium olsonii* (top right), *Penicillium citrinum* (bottom left). The underside of *P. citrinum* plates shows a yellow pigment produced by the fungus (bottom right). The photo was taken after 96 h of incubation at 25 °C. (**b**) Colonies of two *Apergillus* species. The yellow colony is *A. ochraceus* (top), the black colony is *A. niger* (bottom left). The colony on the lower right is *Penicillium* sp. The photo was taken after 6 days of incubation.

Tissue samples consisting of cross-sections made through the stems which supported the inflorescences (Figure 2c) as well as the inflorescences themselves that bore the stalked glandular trichomes on bract tissues were examined by scanning electron microscopy. The cross-sections of the stem revealed the central pith which was surrounded by the pith parenchyma cells (Figure 7a). Also visible were the xylem tissues comprising tracheids and vessel elements. The epidermal tissues made up the outer layer of cells that surrounded the stem. A close-up of the pith parenchyma cells is shown in Figure 7b, with the central pith and surrounding xylem tissues. Higher magnification views of the pith parenchyma cells and vessel elements showed the presence of spore-like structures that were found within these cells (Figure 7c,d). In addition, in some samples, evidence of mycelial growth associated with the xylem vessel elements was observed (Figure 7e). Plating of surface-sterilized inflorescence stem samples onto potato dextrose agar revealed the presence of colonies of *Beauveria*, *Trichoderma*, *Botrytis* and *Penicillium* species (Figure 8). Plating of dried inflorescence tissues prepared as described previously [14] showed the presence of *Penicillium* and *Aspergillus* species (Figure 9).

High magnification views of the stalked glandular trichomes produced within the inflorescences revealed the occurrence of spore-like structures on the surface of the trichome heads (Figure 7f). Many were small circular single-celled spores (Figure 7f), while others were rectangular-shaped and resembled the powdery mildew fungus *Golovinomyces* (Figure 7g). A highly magnified view of the trichome surface showed *Penicillium*-like spores embedded within the resinous material that was secreted from the trichome glands (Figure 7h). NGS analysis of trichome preparations revealed the presence of up to 14 different genera and species (Table 3), most of which are represented in Table 1. The highest incidence was of *Beauveria*, *Fusarium* and *Penicillium* spp. (Table 3). 

## 3. Discussion

Cannabis produced commercially for medicinal and recreational uses in North America and elsewhere is subjected to a range of testing procedures that are aimed to ensure that the product does not contain pesticides, toxins and microbial species that can pose a potential threat to human health following inhalation or ingestion [7]. Among these tests, an assessment of total culturable yeast and mold (TYM) populations present in dried cannabis flowers has garnered considerable attention. The levels of TYM, expressed as colony forming units (cfu) per gram of dried flower, are determined by commercial laboratories that utilize established, but somewhat variable, methods to determine the levels that may be present [8,9,10,11,12,13]. In Canada, the acceptable limit for passing a TYM test is <50,000 cfu/g, which provides a consistent measure of the yeast and mold levels permitted in cannabis products country-wide. The situation is different in the USA, where individual states have mandated different acceptable limits, which can range from 1000 to 100,000 cfu/g [14], depending on the state. This makes a direct comparison of the potential impact of TYM on users of cannabis difficult. For example, would a level of 50,000 cfu/g suggest a greater potential concern for cannabis users compared to a level of 5000 cfu/g ? The actual risks from these different levels of TYM that may be present in cannabis are unknown. In reality, lower levels should provide a greater assurance that the samples pose a reduced risk from mold exposure when compared to higher levels. However, the greatest unknown factor at present that impacts the interpretation of TYM levels for safety assessment of cannabis is a poor understanding of which specific fungal or yeast species are contributing to the culturable TYM that are recovered on agar media. Several previous studies have identified and confirmed the specific fungal/yeast species that can contribute to the TYM count [15,16,17,18,19,20]. These types of studies need to be conducted utilizing a broader range of samples from different growing regions and under varied production environments to establish a base-line of TYM. This information is necessary to be able to utilize TYM counts as a consistent indicator of product safety for cannabis users. Currently, TYM numbers reflect a variable and unknown population around which safety and quality assurances are established.

TYM levels in cannabis have been reported to be affected by the cannabis genotype being cultivated, by environmental conditions during production, and by post-harvest handling practices [14]. In addition, drying conditions that achieve a water activity of 0.65–0.7 in the cannabis tissues have a greater probability of containing lower viable TYM counts [22,23]. Drying is a well-established process that reduces microbial levels in many plant-derived products, including herbs and spices, thereby extending their shelf-life and enhancing quality [24,25,26]. The low water activity prevents further proliferation and growth of these molds. Generally, a water activity of 0.9 or higher is required for extensive growth of resident fungi [27,28], which may occur during the final curing stages of cannabis if moisture level is not adequately managed. Regardless, certain molds, in particular species of *Penicillium* and *Aspergillus*, are reported to survive despite the drying conditions and may still be viable in the final cannabis product.

In the present study, a whole genome sequencing approach was used by employing NGS to determine which fungal and yeast species were present in greenhouse-grown cannabis inflorescences. The composition of this mycobiome was separated into primary and secondary populations, based on the frequency of fungal reads. In the primary populations (represented by reads greater than 1% of the total fungal reads), 26 species of yeast and mold were identified. The genera with the highest frequency of occurrence (each representing >20% of total reads depending on the sample) included *Alternaria*, *Botrytis*, *Fusarium* and *Penicillium*, which are previously reported as facultative pathogens on cannabis [1,2]. In addition, non-pathogenic or saprophytic genera present were *Aureobasidium*, *Metarhizium* and *Trichoderma*, which also constitute beneficial fungi and yeasts with biological control activity against plant pathogens [29,30,31,32]. Other genera in the primary population (represented by reads between 1 and 10% of total reads) included *Aspergillus*, *Candida*, *Phycomyces*, *Rhizopus* and *Rhizophagus*. Most of these genera would be considered as saprophytic fungi or yeasts, with the exception of *Aspergillus* and *Rhizopus*, which are secondary pathogens on plants, while *Rhizophagus* is a mycorrhizal fungus [33] which has been reported to improve growth of cannabis seedlings [34]. The diversity of the mycobiome suggests that TYM populations in cannabis inflorescences can result from both pathogenic infection of the plants as well as from secondary colonization by fungi or yeasts from environmental sources, or following application of biocontrol fungi for disease management [35,36].

A tissue source not previously studied for TYM presence that was included for NGS analysis was the inflorescence stems that supported the developing inflorescences and comprised the vascular tissues within the stem. Within these tissues, scanning electron microscopy of stem sections demonstrated the presence of spore-like structures and mycelium of putative fungal species within pith parenchyma cells and the xylem vessel elements. NGS analysis identified the predominant genera associated with the inflorescence stems (based on highest percentages of fungal reads) to be *Penicillium*, *Fusarium* and *Rhizophagus*. The inflorescence stems may provide protection against environmental extremes and enhance survival of these fungi, in addition to potentially supporting endophytic spread. Other fungi associated with the inflorescence stems included *Alternaria*, *Aspergillus*, *Trichoderma* and the yeast *Candida*; these genera were also present within the surrounding inflorescence tissues. During the preparation of cannabis samples for TYM testing in commercial laboratories, whole inflorescence samples are ground in buffer solution and generally incubated prior to analysis by dilution-plating [12]. The grinding process would include the fungi resident within the stem tissues, potentially enhancing overall TYM counts. 

In an effort to establish the potential sources of the fungal and yeast genera present in cannabis tissues, samples of the growing medium (mostly comprising coco fiber substrate) were analyzed by NGS. A number of the species present in inflorescence tissues were present in the growing substrate. Furthermore, several of these genera (*Alternaria*, *Botrytis*, *Candida*, *Fusarium*, *Penicillium* and *Rhizophagus*) were also found to be present in a limited number of seed samples that were included for NGS analysis, illustrating the potential for seed-borne transmission of some of these endophytic species. Previous studies have confirmed the occurrence of a range of bacterial and fungal species on or within cannabis and hemp seeds [20]. Many of these species had beneficial roles in providing protection to the germinating seed against pathogens and improving growth, while others were potentially detrimental and included plant pathogens [20]. Their presence in the seed could have resulted from endophytic spread of the associated microbes within the vascular tissues into the seed. A microbiome study of seedlings of several cannabis genotypes has shown that a number of fungi, including *Penicillium* spp., could be recovered from seeds that were plated onto agar medium [37].

A comparison of fresh inflorescence tissues with dried tissues by NGS illustrated that drying reduced the frequency of fungal reads of many genera, with a greater impact on the occurrence of *Trichoderma* compared to *Penicillium* and *Rhizophagus*, which were less affected. Biological control products containing *Trichoderma* and *Beauveria*, when applied during flower development [35,36], resulted in enhanced levels in fresh inflorescences, with a lower carry-over into dried samples. *Trichoderma* and *Beauveria* species differ in their response to drying and tolerance to desiccation and high temperatures [38,39,40]. In three dried inflorescence samples with >50,000 cfu/g (failed samples), *Penicillium*, *Fusarium*, *Botrytis* or *Beauveria* spp. were predominant and made up 27–81% of total fungal reads, depending on the sample. Colonization of cannabis inflorescences by mycelium of *Fusarium* and *Botrytis* therefore can lead to failure to pass TYM tests. *Rhizophagus* produces resilient spore structures that can survive desiccation [41] but it is difficult to culture in vitro and it would not contribute to TYM levels as currently measured. The fungal and yeast species which have a high tolerance to drying and that are able to subsequently grow on culture media would therefore represent the highest probability of being recovered in TYM testing. These include *Penicillium*, *Fusarium*, *Botrytis* and *Beauveria* spp.

It is noteworthy that species of *Aspergillus* that could potentially be of concern to human health when present on cannabis samples were not detected in this study. Of the four species detected by NGS, none have been associated with the risk for aspergillosis [7]. One species (*A. terreus*) has been previously recovered from coco coir growing substrate and was shown to be inhibitory to the development of root-infecting plant pathogens [42,43]. In many agricultural crops, the environmental conditions for *Aspergillus* contamination of plant-derived products, including corn, wheat and peanuts, where contamination from fungal-associated mycotoxins is common, generally involve hot and humid climates with moisture from excessive humidity or rainfall near harvest [44]. Such conditions are not common under greenhouse conditions where cannabis is grown legally, although they can occur on field-grown hemp [45], resulting in fungal invasion of the inflorescences. Rapid drying and proper storage of cannabis inflorescences would also preclude post-harvest contamination by *Aspergillus* species that could otherwise raise concerns for mycotoxin contamination. Ochratoxin A production by *Aspergillus* species on cereal grains was optimal at a water activity (*a*_w_) of 0.98 and was minimal at *a*_w_ of 0.83–0.87 [46]. These findings suggest that licensed greenhouse-cultivated cannabis has a low risk for *Aspergillus* contamination and associated mycotoxins. This may not be the case for contamination of inflorescences by *Alternaria* and *Fusarium* species, where mycotoxin production has been shown to occur under conditions where mycelial growth is prolific under conducive environmental conditions [47]. The frequency of such events occurring in indoor growing environments, including greenhouses, is unknown. Infection of inflorescences by *Botrytis* during cultivation of cannabis [36] may also enhance TYM counts, but any additional risk from fungal toxins has not been reported. These findings suggest that the fungal genera that can contribute to a failure to pass TYM limits imposed by regulatory agencies may involve *Penicillium*, *Botrytis*, *Fusarium* and *Alternaria*. These fungi grow well on agar media and would be recovered in current TYM testing. In contrast, toxigenic *Aspergillus* species appear to be less commonly present.

In addition to the inflorescence stem tissues, scanning microscopy was also conducted on the bract tissues bearing stalked glandular trichomes on dried cannabis samples. Close examination revealed the presence of different types of fungal-like spore structures that had adhered to the surface of the glandular heads. The secretion of resinous materials when the trichomes approach maturity at harvest [48] can increase opportunities for fungal spores to be trapped in the resin. NGS analysis of extracted trichome samples confirmed the presence of a range of wind-disseminated genera, which could have resulted from spores that may contribute to TYM. In particular, spores of *Beauveria*, *Fusarium*, *Penicillium* and *Trichoderma* were present on trichome surfaces. However, the viability of these spores is undetermined and further testing is needed to determine if they could contribute to the TYM counts. The current TYM testing methods in which species identification is not included may therefore cause products which contain high levels of biocontrol fungi and other species that may be considered to be less harmful to cannabis users to fail. 

The most frequently occurring fungal genus on the cannabis samples assayed in this study was *Penicillium*, with the species *P. chrysogenum* and *P. citrinum* being the most prevalent. Despite the widespread occurrence reported in this study and in previous studies [15,17], *Penicillium* has not received the same attention as *Aspergillus* for an evaluation of its ability to cause potential harm to cannabis users. Its ubiquitous presence in the cannabis samples included in this study suggests that it may be one of the main causes of cannabis samples failing to pass TYM tests. It is easy to recover on agar medium and the antibiotic-producing properties of both species reported here would likely inhibit the growth of other fungal and yeast species. *Penicillium* species were shown to be one of the most common causes of surface mold contamination on dried cannabis [15], and they are found on cereals, dried herbs, cheeses and a range of other foods [49,50,51]. They are present in soil and are commonly found in cool damp areas that include buildings and storage facilities and are a common cause of indoor mold allergies [52]. On a broader scale, the agricultural, biotechnological and pharmaceutical applications of *Penicillium* species that are endophytes of medicinal plants have been reviewed, illustrating the diversity of roles of this genus [53]. Among these, antimicrobial production was the most important role which inhibited growth of a range of bacterial and fungal species. Accordingly, there are numerous reports highlighting the beneficial properties of *P. chrysogenum* as a biological control agent that can reduce disease development and improve plant growth on a number of plant species [54,55,56,57]. *Penicillium* species therefore appear to have contrasting roles in cannabis tissues. Whether the reported beneficial properties are exhibited within cannabis inflorescence tissues is presently unknown.

A recent survey of cannabis flower samples and concentrates for mycotoxin presence in 11 US states revealed that the aflatoxins B1, B2, G1 and G2 reported to be produced by *Aspergillus* species were rarely detected in more than 440,000 samples tested, indicating that consumer exposure to *Aspergillus* and its corresponding regulated mycotoxins was likely rare in legal markets [58]. Ochratoxin A, however, which is known to be produced by *Penicillium* species in addition to *Aspergillus* species [59], was detected at rates of 3.28% in flower and 8.32% in concentrates across all testing laboratories surveyed. These levels did not exceed regulatory action limits. The results from the present study appear to support Boyar et al.’s findings [58], suggesting that certain ochratoxin A-producing *Penicillium* and *Aspergillus* species may be a more common source of contaminants on cannabis products. *Aspergillus ochraceus* and *A. niger* (Figure 9) have been previously reported to occur on cannabis inflorescences [1,14,15] and are known to produce Ochratoxin A [60,61,62]. 

There are a number of previous studies that have reported fungal species of potential concern in cannabis samples using molecular analyses. These have shown that species of *Aspergillus*, *Penicillium* and *Fusarium* can be identified using PCR-based techniques with specific primers and by whole genome sequencing [16,17,18,19,20,47], supporting the results from the present study. The current study employed a whole-genome sequencing approach, which, while providing insight into the fungal and yeast species present, only provided a measure of relative abundance and not of actual levels of the species. The percentage of fungal reads in most samples was relatively low, equating to around 0.03–0.5% of eukaryotic DNA, of which 98–99% constituted cannabis DNA that was excluded in the analysis. The actual amount of fungal or yeast presence (as colony-forming units) cannot be estimated and in cases where reads constituted less than 1% of total fungal DNA (Table 2), it may be represented by a few spores and/or limited mycelium that was based on small amounts of DNA. It is also not known what proportion of the fungal reads were dead vs. living cells at the time of sampling. Since current yeast and mold analysis requires living cells to be cultured on agar media, these results may not be directly comparable. However, the NGS approach reveals the specific species that were present in the tissue samples and their possible origins. Some of the secondary or minor species could have originated from spores adhered to the surfaces of trichome heads from air-borne inoculum for which the viability is unknown. 

**Figure 10:**
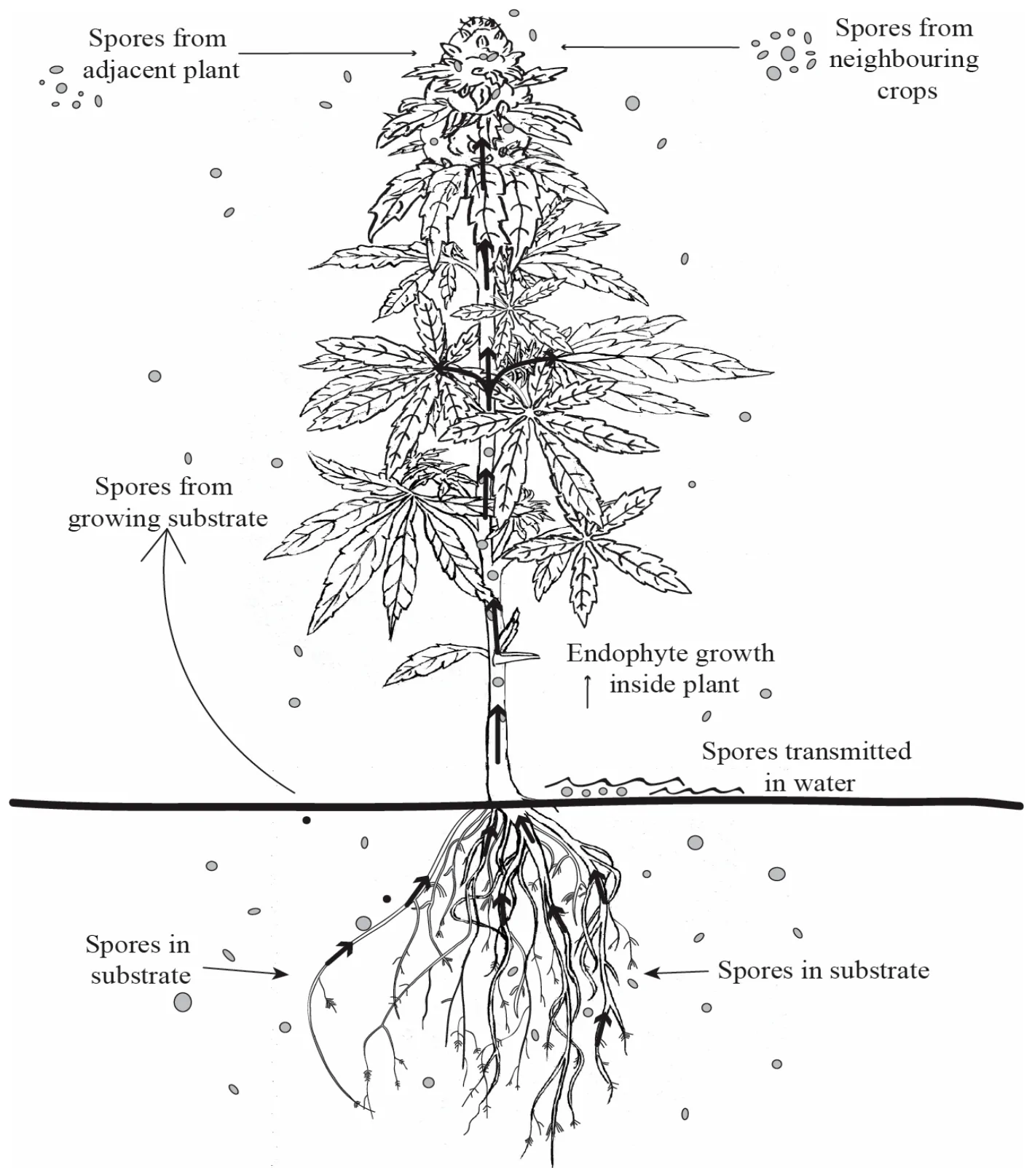
The different avenues through which total yeast and mold (TYM) may develop on cannabis inflorescences. They include aerially disseminated spores from adjacent plants or crops, the growing substrate, water, and endophytic spread within the plant.

A summary of the different avenues through which TYM may occur on cannabis inflorescences is presented in Figure 10. They include aerially disseminated spores from adjacent plants or crops, the growing substrate, water, and potentially endophytic spread within the plant. The levels of yeast and mold that eventually develop would be influenced by the genotype of cannabis being grown, as some are more conducive to fungal colonization than others [14], and by the environmental conditions under which the plants are grown. In particular, high humidity with poor air circulation would enhance the probability for more extensive colonization of the tissues [14]. 

In an effort to determine the possible origins of the secondary species and their potential to contribute to TYM counts, a survey of their roles in the environment was conducted from the current literature. The findings are presented in Table 2 and show that up to 50% of the fungal and yeast species were saprophytes and secondary decomposers commonly associated with organic matter and can be found in soil. A number were reported to be plant pathogens and endophytes. Interestingly, none of the reported pathogens are known to infect cannabis and are previously reported from non-cannabis hosts, including cereal crops. This suggests their presence on cannabis may be incidental. They were detected on both fresh and dried inflorescences. However, since the frequency of occurrence of the secondary species was very low (representing <1% of total fungal reads), they are not considered to be significant in contributing to the total yeast and mold counts. In addition, since the detection was based on the presence of DNA sequences, these species may occur as dead propagules following drying, particularly for the yeast species that are sensitive to desiccation. In one instance, a species of *Tuber* that forms mycorrhizal associations with specific hosts was found. This species is cultivated commercially in locations in close proximity to cannabis cultivation but the method of spread is undetermined [63]. The two lichen species detected in the inflorescences are also reported to be present in British Columbia where this study was conducted [64] and they could have their origins in the growing substrate or from the external environment. Lastly, the presence of human DNA in a number of dried samples (but not in fresh samples) suggests contamination from post-harvest handling of the inflorescences. The next-generation sequencing method can detect low amounts of DNA originating from human skin cells or fragments of hair.

## 4. Conclusions

The results from this study have identified a range of fungal and yeast species which were present in greenhouse-cultivated cannabis inflorescences using a whole genome sequencing approach. While a direct comparison between TYM plate counts (the totality of culturable species) and those identified using next generation sequencing methods (detection of all probable species) cannot be made, the results show both the diversity and prevalence of potential microbial colonizers of the inflorescences. In particular, they show that *Penicillium* spp. is a major component of this colonization (Figure 9), both pre-harvest and post-harvest. This perhaps necessitates a further evaluation of its potential to cause harm, either directly or through mycotoxin production. Toxigenic *Aspergillus* species were found to be much less common in legal greenhouse cannabis production. The contribution of stem-associated populations of yeast and mold to culturable TYM counts needs to be assessed further. An understanding of the specific species which have the potential to cause harm may be more informative than the total number of culturable species, which can include those considered to be less harmful to humans. High yeast and mold levels are undesirable as they reflect cannabis production under environments that promote colonization, leading to an inferior product. However, do current TYM testing methods provide a quality assessment which may not correspond to a safety assessment? Additional studies are warranted to evaluate the interpretation of TYM testing in cannabis for safety and quality control by including an identification of the species that are most prevalent and assessing their potential for harm. In the current study, the most commonly identified genera (*Penicillium*, *Alternaria*, *Fusarium* and *Botrytis*) are not included in the species of concern under the current regulatory framework in place for cannabis testing. 

## 5. Materials and Methods

### 5.1. Sample Sources

The cannabis inflorescence samples included in this study were obtained from greenhouse-grown plants (Figure 1a–c) cultivated either using a hydroponic system (five genotypes) or from organic production (two genotypes). The genotypes selected were those that were in production at the time of sampling. All samples originated from the same greenhouse environment within these production systems and were collected at several different times during 2024. From each plant selected for sampling, 4–5 inflorescences were obtained and combined to provide one bulk sample. At some sampling times, application of biological control products that included *Trichoderma* and *Beauveria* were made to the flowering plants according to previously described application methods [35,36]. All collected samples were used fresh or following drying according to commercial practice (Figure 1d) to achieve a final moisture content of around 12–14% (Figure 1e). A total of 24 inflorescence samples were subjected to NGS analysis as described below, each represented by a single replicate. The sample descriptions can be found at https://kannapedia.net/strains and in Supplementary Table S1. Additional samples included for NGS analysis included seed and the growing medium.

In addition to the whole inflorescences, samples of stems supporting the inflorescence structure (Figure 2) were dissected from the hydroponically-grown inflorescences. Trichome preparations were also included from one cannabis genotype by taking dried inflorescence samples and gently crushing them by hand and then sifting the material through a series of five stacked sieves (1.18 mm, 600 µm, 250 µm, 150 µm and 75 µm sizes). The dried material retained on the 150 µm sieve consisted of trichome stalks and glandular heads (Figure 3a,b). All samples were used for DNA extraction as described below. 

### 5.2. Next-Generation Sequencing

Tissues (50–100 mg) from each sample were homogenized using a mortar and pestle with liquid nitrogen into a fine powder to ensure thorough cell disruption. DNA extraction was carried out using the Qiagen DNeasy Plant Mini Kit (cat. 69104, QIAGEN GmbH, Hilden, Germany) following the manufacturer’s protocol. DNA quality and quantity were assessed using a NanoDrop spectrophotometer (Thermo Fisher Scientific, Waltham, MA, USA), while DNA integrity was confirmed by electrophoresis on a 1% agarose gel. The DNA samples were forwarded to Medicinal Genomics (https://medicinalgenomics.com/) for high throughput metagenomic sequencing using their in-house protocols. Briefly, libraries were made with Watchmaker DNA Library Prep Kit with Fragmentation according to the manufacturer’s protocol (https://www.watchmakergenomics.com/products/dna-ngs-solutions/dna-lpk.html). Dual index Illumina primers from New England Biolabs Inc. (Ipswich, MA, USA) (http://www.neb.com) were incorporated into the libraries. Quality and quantity of the libraries were measured on Agilent tapestation (https://www.agilent.com) using High Sensitivity D5000 screen tapes. Equal moles of libraries were pooled together and sequenced on the Illumina NovaSEQ sequencing platform (https://www.illumina.com). The DNA sequences were aligned to the maternal cannabis genotype ‘Jamaican Lion’ reference genome using DRAGEN unified genotyping platform (Dynamic Read Analysis for GENomics) (HostSoftware, Version 3.8.4) (https://www.illumina.com). Reads were trimmed with fastp [65] for metagenomics analysis. The reads that did not map to the ‘Jamaican Lion’ reference genome (inclusive of microbial and other sequences) were retained and used in the metagenomics analysis in the present study using the Kraken and Braken sequence alignment software [66]. Braken (Bayesian Re-estimation of Abundance with KrakEN) is a highly accurate statistical method that computes the abundance of species in DNA sequences from a metagenomics sample. The Kraken database used was a custom database built in 2023 using the default options and capping off the memory at 16 Gb. Several additional sequences, including fungal sequences, were added to the defaults to create a more comprehensive database to provide identification of potential fungal species of relevance to cannabis production. Species identifications from the Kraken database were not verified by specific sequence comparisons following PCR for each individual species. Taxonomic outputs were visualized using Krona plots to enable interactive and hierarchical visualization of microbial diversity. Kraken tools were used to generate these files using the KronaTools [67] utility ktImportText (https://nf-co.re/modules/krona_ktimporttext). A custom script was developed to convert these Krona html files into a tab-delimited file to show the total numbers of reads and the percentage of reads that were classified into each taxonomic category represented in the Krona plots. The FASTA files and Krona plots facilitated a detailed exploration of microbial community structure and differences across the cannabis samples that were included for analysis. The results are presented as total fungal reads as well as a percentage of total fungal reads in a given sample. All of the original data sets are available at: https://kannapedia.net/strains. This depository also includes a large number of additional samples that were not a part of this study. The samples included in this study are identified by the principal author’s name and are summarized in Supplementary Table S1. 

### 5.3. Sample Analysis

To ensure reproducibility of the results presented, several rounds of NGS were conducted utilizing different samples of the same cannabis genotypes collected at different time points. The data from the repeated samples were averaged since time of sampling was not found to be a variable and are presented in Table 1 and Table 2. The results are expressed with regard to each specific species identified as a percentage of the total fungal and yeast reads. The data in Table 1 and Table 2 are presented based on the frequency of occurrence, with fungal and yeast species with more than a 1% frequency of total reads shown in Table 1 and those with less than 1% frequency shown in Table 2. The identity of each of the species shown was not verified using specific PCR for the respective species and sequence comparisons.

### 5.4. Scanning Electron Microscopy

Samples of inflorescence tissues and stems supporting the inflorescence were obtained for scanning microscopy. The stem samples were sectioned by hand using a sharp blade to provide a cross-sectional view of the stem. Pieces of both tissue types measuring 0.5 cm^2^ were adhered to a stub using a graphite-water colloidal mixture (G303 Colloidal Graphite, Agar Scientific, Rotherham, UK) and Tissue-Tek (O.C.T. Compound, Sakura Finetek, Alphen aan den Rijn, The Netherlands). The samples were submerged in a nitrogen slush for 10–20 s to rapidly freeze them. After freezing, the sample was placed in the preparation chamber of a Quorum PP3010T cryosystem attached to a FEI Helios NanoLab 650 scanning electron microscope (4D Labs., Simon Fraser University, Burnaby, BC, Canada). The frozen samples were sublimed for 5 min at −80 °C, after which a thin layer of platinum (10 nm thickness) was sputter-coated onto the samples for 30 s at a current of 10 mA. The samples were moved into the SEM chamber and the electron beam was set to a current of 50 pA at 3 kV. Images were captured at a working distance of 4 mm, at a scanning resolution of 3072 × 2207 collected over 128 low-dose scanning passes with drift correction.

## Figures and Tables

**Table 1:** The primary fungal and yeast genera and species present in cannabis inflorescences as determined by whole genome sequencing. Total reads are the means from both fresh and dried inflorescences representing four genotypes. The range reflects the variation in total reads between the samples. Reads shown in **bold** reflect highest incidence of reads detected among the samples.

Fungal/Yeast Genus	Species Present	Mean and Range of Total Reads (%)	Detection from Other Sources
*Alternaria* *Aspergillus* *Aureobasidium* *Botrytis* *Candida* *Fusarium* *Metarhizium* *Penicillium* *Phycomyces* *Rhizopus* *Trichoderma* *Rhizophagus*	*A. alternata* *A. infectoria* *A. glaucus* *A. melleus* *A. sclerotioniger* *A. terreus* *A. pullulans* *B. byssoidea* *B. cinerea* *C. albicans* *C. jutengensis* *C. subhashi* *C. tropicalis* *F. fujikori* *F. graminearum* *F. oxysporum* *F. proliferatum* *M. acridum* *P. brevicompactum* *P. citrinum* *P. chrysogenum* *P. lagena* *P. blakesleannus* *R. stolonifer* *T. harzianum* *R. irregularis*	(1.0)—1.5—(**29.0**) (1.0)—2.3—(13.0) (1.0)—2.2—(3.0) (2.0)—2.9—(6.0) (1.0)—1.2—(2.0) (1.0)—2.2—(3.4) (1.0)—4.6—(**20.1**) (2.0)—3.2—(6.0) (2.0)—7.6—(**28.0**) (1.5)—2.0—(3.0) (1.0)—1.4—(3.0) (1.0)—1.3—(3.0) (1.0)—2.1—(3.0) (1.0)—4.1—(11.0) (1.0)—2.4—(7.0) (1.0)—7.6—(**40.0**) (1.0)—2.1—(4.0) (1.1)—7.0—(**20.0**) (1.0)—7.4—(8.0) (2.0)—4.8—(**15.0**) (3.0)—18.4—(**56.0**) (1.0)—1.7—(3.0) (1.1)—3.3—(6.8) (1.0)—3.0—(4.0) (2.0)—2.8—(**46.0**) (1.0)—4.0—(7.0)	Seed Growing medium Growing medium Growing medium ---- Growing medium Growing medium --- Seed Seed --- --- --- Seed, growing medium Growing medium Growing medium Growing medium -- Seed, growing medium Seed, growing medium -- Growing medium Growing medium Growing medium Seed, growing Medium

**Table 2:** The secondary fungal, yeast and other genera and species present in cannabis inflorescences as determined by whole genome sequencing. Incidence of these species was represented at a frequency of <1% of total reads.

Fungal/Yeast/Other Genus and Species	Source and/or Role in the Environment	Presence (+) or Absence (−) in Fresh Inflorescences ^a^	Presence (+) or Absence (−) in Dried Inflorescences ^a^
*FUNGI*			
*Beauveria bassianae*	Insect pathogen/biocontrol fungus	+	+
*Botrysphaeria* sp.	Plant pathogen	+	−
*Chaetomium globosum*	Saprophyte and secondary pathogen	+	+
*Colletotrichum acutatum/*	Plant pathogen/endophyte	+	+
*C. gloeosporiodes*	Plant pathogen/endophyte	+	+
*Diaporthe* sp.	Plant pathogen/endophyte	+	−
*Mucor mucedo*	Saprophyte and decomposer	+	
*Neurospora* sp.	Saprophyte and decomposer	+	+
*Pestalotiopsis* sp.	Plant pathogen/endophyte	+	+
*Pyricularia pennistigena*	Plant pathogen	+	+
*Ramularia collo-cygni*	Plant pathogen	+	+
*Rhizopus microspores*	Saprophyte and decomposer	+	+
*Scedosporium apiasperum*	Plant pathogen	+	+
*Sordaria* sp.	Saprophyte and decomposer	+	+
*Tuber melanosporum*	Mycorrhizal fungus	+	+
*Verticillium* sp.	Plant pathogen/endophyte	+	+
*YEASTS*			
*Blastomyces* sp.	Saprophyte and decomposer	+	+
*Cokeromyces recurvatus*	Saprophyte and decomposer	+	+
*Cryptococcus* sp.	Saprophyte and decomposer	+	+
*Paraccociodes brasiliensis*	Saprophyte and decomposer	+	−
*Saccharomyces cerevisiae*	Saprophyte and decomposer	+	−
*Sporothrix* sp.	Saprophyte and decomposer	+	+
*Trichosporon asahi*	Saprophyte and decomposer	+	+
*OTHER*			
*Endocarpon pusillum (lichen)*	Saprophyte and decomposer	+	+
*Letharia columbiana (lichen)*	Saprophyte and decompose	+	+
*Homo sapiens*	Human	−	+

^a^ Based on molecular detection from whole genome sequencing of samples.

**Table 3:** Fungal and yeast genera and species, each representing >1% of total fungal reads, associated with trichome preparations from inflorescences of cannabis plants.

Genus and Species	Frequency of Occurrence (% of Total Reads)
*Aspergillus melleus*	1.0
*Alternaria alternata*	4.0
*Beauveria bassianae*	26.0
*Botrytis cinerea*	1.5
*Candida albicans*	2.0
*Fusarium proliferatum*	1.0
*Fusarium oxysporum*	16.0
*Golovinomyces ambrosiae*	1.5
*Penicillium brevicompactum*	3.5
*Penicillium chrysogenum*	19.0
*Penicillium citrinum*	12.0
*Penicillium lagena*	1.0
*Saccharomyces cerevisiae*	1.0
*Trichoderma harzianum*	6.5

## Data Availability

All data generated or analyzed during this study are included in this published article.

## Supplementary Materials

The following supporting information can be downloaded at: https://www.mdpi.com/article/10.3390/plants15213339/s1, Table S1: Summary of the cannabis inflorescence samples included in this study.
