# Supplementary material for: Next-Generation Sequencing Reveals the Total Yeast and Mold Species Present in Greenhouse-Grown Cannabis Inflorescences—Implications for Quality Control
Source: Plants (Basel). 2026 Oct 28;15(21):3339. doi: 10.3390/plants15213339 (PMC13765965; doi:10.3390/plants15213339)
Supplement: Supplementary file 1 [file plants-15-03339-s001.zip › plants-4552309-supplementary.pdf]

Supplementary Table S1. Summary of the cannabis inflorescence samples included in this study that are also shown on : https://kannapedia.net/strains

Sample ID (RSP Number)	Genotype	Fresh/Dried	Conventional /Organic	Date of sampling
PG (13087)	Pink Kush	Fresh	Conventional	February 7 2024
JFG (13085)	Powdered Donuts	Fresh	Conventional	February 7 2024
PG (13088)	Pineapple God	Fresh	Conventional	February 7 2024
CDSD (13084)	Cherry Do-Si-DO	Fresh	Conventional	February 7 2024
DB (13083)	Donnie Burger	Fresh	Conventional	February 7 2024
BF (13086)	Blueberry Funk	Fresh	Conventional	February 7 2024
HM buds (13128)	Hollywood Mac	Fresh	Organic	June 2 2024
SHL buds (13131)	Super Lemon Haze	Fresh	Organic	June 2 2024
BCP bud (13137)	Berry Cream Puff	Fresh	Conventional	June 5 2024
BCP drying (13118)	Berry Cream Puff	Dried	Conventional	April 24 2024
Blch buds (13134)	Black Cherry	Fresh	Conventional	June 2 2024
DB dried (13119)	Donnie Burger	Dried	Conventional	April 24 2024
El Gordo dried (13122)	El Gordo	Dried	Conventional	April 24 2024
EGf buds(13148)	Electric Grapefruit	Fresh	Conventional	June 5 2024
PG buds (13151)	Pineapple God	Fresh	Conventional	June 5 2024
PK Buds (13153)	Pink Kush	Fresh	Conventional	June 5 2024
PK drying (13124)	Pink Kush	Dried	Conventional	April 24 2024
PK dried (13123)	Pink Kush	Dried	Conventional	April 24 2024
T20 drying (13125)	T20	Dried	Conventional	April 24 2024
DP dried (13143)	Duct Tape	Dried	Conventional	April 24 2024
DB dried (13220)	Donnie Burger	Dried	Conventional	April 24 2024
C47 (13221)	C47	Dried	Conventional	April 24 2024
Elec. Grp. Drying (13121)	Electric Grapefruit	Dried	Conventional	April 24 2024
